# Trehalose Restrains the Fibril Load towards α-Lactalbumin Aggregation and Halts Fibrillation in a Concentration-Dependent Manner

**DOI:** 10.3390/biom11030414

**Published:** 2021-03-11

**Authors:** Sania Bashir, Ishfaq Ahmad Ahanger, Anas Shamsi, Mohamed F. Alajmi, Afzal Hussain, Hani Choudhry, Faizan Ahmad, Md. Imtaiyaz Hassan, Asimul Islam

**Affiliations:** 1 Centre for Interdisciplinary Research in Basic Sciences, Jamia Millia Islamia, Jamia Nagar, New Delhi 110025, India; sania_kim28@yahoo.co.uk (S.B.); ahangerishfaq123@gmail.com (I.A.A.); anas.shamsi18@gmail.com (A.S.); fahmad@jmi.ac.in (F.A.); mihassan@jmi.ac.in (M.I.H.); 2 Department of Chemistry, Biochemistry and Forensic Science, Amity School of Applied Sciences, Amity University, Haryana 122413, India; 3 Department of Pharmacognosy, College of Pharmacy, King Saud University, Riyadh 11451, Saudi Arabia; malajmii@ksu.edu.sa (M.F.A.); afzal.hussain.amu@gmail.com (A.H.); 4 Department of Biochemistry, Faculty of Science, King Abdulaziz University, Jeddah 21589, Saudi Arabia; hchoudhry@kau.edu.sa

**Keywords:** protein aggregation, trehalose, spectroscopy, transmission electron microscopy, molecular docking

## Abstract

Protein aggregation and misfolding are some of the most challenging obstacles, customarily studied for their association with amyloid pathologies. The mechanism of amyloid fibrillation development is a dynamic phenomenon involving various factors such as the intrinsic properties of protein and the physical and chemical environmental conditions. The purpose of this study was to see the thermal aggregation profile of alpha-lactalbumin (α-LA) and to delineate the effect of trehalose on its aggregation profile. α-LA was subjected to thermal aggregation at high concentrations. UV-Vis spectroscopy, a turbidity assay, intrinsic fluorescence, Rayleigh scattering and a thioflavin T (ThT) assay explained the steady outcomes that 1 M trehalose repressed α-LA aggregation in the most effective way followed by 0.75 M and 0.5 M and to a significantly lesser degree by 0.25 M. Multi spectroscopic obser Sania **Bashir** ations were further entrenched by microscopy. Transmission electron microscopy confirmed that in the presence of its higher concentration, trehalose hinders fibril development in α-LA. In vitro studies were further validated by in silico studies. Molecular docking analysis indicated that trehalose occupied the binding pocket cavity of α-LA and offered several significant interactions, including H-bonds with important residues. This study provides a platform for trehalose in the therapeutic management of protein aggregation-related diseases.

## 1. Introduction

Several neurodegenerative diseases leading to amyloid fibrils and plaques are unambiguously associated with the intracellular aggregation of proteins [1,2]. Notwithstanding, this fibrillation process experiences several events including halfway misfolding followed by the development of oligomers, protofibrils and long-run fibrils. Alpha-lactalbumin (α-LA), a Ca^2+^ metallo binding [3] milk protein, is a constituent of whey proteins. Whey proteins, including α-LA, are exposed to heat during the process of pasteurization. Heating extensively leads to the aggregation and gel development of proteins with both favorable and non-favorable effects depending upon the objective of utilizing protein ingredients [4]. Recently, there has been an emerging interest growing to utilize conceivable heat treatments to guarantee and maintain protein’s nutritional value and longevity. Thus, the choice of an osmolyte, which potentially acts as a molecular aggregation blocker and contributes towards preventing aggregation in heat-sensitive proteins such as α-LA, maybe one of the novel approaches. There are several reports in the literature of folding and unfolding studies leading to aggregation, including α-LA [5,6,7]

Osmolytes, which are naturally occurring chemical chaperones, are intracellularly accumulated to adapt heat and osmotic pressure. Their importance in maintaining protein stability and folding has been previously acknowledged [8,9,10,11]. The role of osmolytes in regulating the aggregation pathway of protein is in progress and feebly comprehended. Among various osmolytes, trehalose has been visualized considerably for its broad range relevance [12] with a generally regarded as safe (GRAS) value status [13]. However, various speculations have been declared to clarify its adjustment behavior [14,15,16,17,18,19,20]. One of the speculations is that its machinery of stabilization is intricate and cannot be attributed exclusively to one theory [21]. Various studies have detailed that sucrose, trehalose and proline decrease/restrain aggregation in model proteins such as lysozymes and α-LA or therapeutic proteins such as α-synuclein and insulin, which cause neurodegenerative diseases [22,23,24]. Even though osmolytes are generally not protein-specific, nonetheless, in specific cases, osmolytes may destabilize or aggregate a particular protein [22]. On the other hand, many osmolytes are known to stabilize proteins, and this stabilization is protein-dependent [22,25]. Subsequently, there is a requirement for a selective osmolyte treatment because different osmolytes affect proteins differently. According to the water replacement theory, sugars help proteins maintain their native structure by facilitating H-bonds formation between proteins and water molecules [26].

In the current research, we explored the effect of trehalose on α-LA aggregation. This study was motivated by our earlier work that showed saccharides as potential osmolytes interceded with the aggregation of several proteins [27]. Proteins are stabilized in the presence of trehalose in the native state and help rejuvenate denatured proteins by maintaining steady non-native states [12,28]. Several hypotheses have been proposed to clarify this stabilization by trehalose [12]. The vitrification hypothesis proposes that trehalose forms a cocoon-shaped matrix that protects proteins from stress conditions. Trehalose is a genuinely exceptional sugar as it does not have a reducing hydroxyl end group, being made out of two glucose molecules associated through α,α-1,1 linkage (Figure S1). Table S1 lists its various physical and chemical properties. This osmolyte has incredible capacities to ensure the natural structures of proteins during dehydration and fills in as a bio-protectant against different stresses; for example, osmotic shock, desiccation, freezing or heat [29,30].

Trehalose has a significant impact on protein misfolding disorders such as Alzheimer’s disease [31] and Huntington’s disease [32,33]. This osmolyte reduces aggregation by driving aggregation towards an off-pathway amorphous aggregation formation. Trehalose as a potential osmolyte could also inhibit the aggregation reported by Liu et al. [31] and others [34,35,36] in various neurological disorders where responsible peptides such as Aβ42 and Aβ40 dissolved their aggregates, causing Alzheimer’s disease. Promising results were also seen in Huntington’s disease [37] where a competent inhibitor of poly-glutamine was reported to be aggregated by Tanaka et al. [33]. Trehalose could likewise repress prion aggregation [38,39]. Few reports exist representing its ability to significantly contribute to Parkinson’s disease PD therapy as well [40,41,42].

Consequently, in this present investigation, we have utilized biophysical and computational experiments to obtain better and more profound insights into how trehalose’s presence prevents aggregation in globular proteins. Trehalose was chosen as it has been demonstrated to be the best molecular chaperone against thermal stress [15]. Trehalose masks the beginning of protein aggregation [23]. The results from this study might help to design restorative drugs against Alzheimer’s and Parkinson’s.

## 2. Results and Discussion

### 2.1. UV-Vis Spectroscopy

#### Thermal Transition Curve of α-LA in the Presence of a Varying Concentration of Trehalose

**Figure 1:**
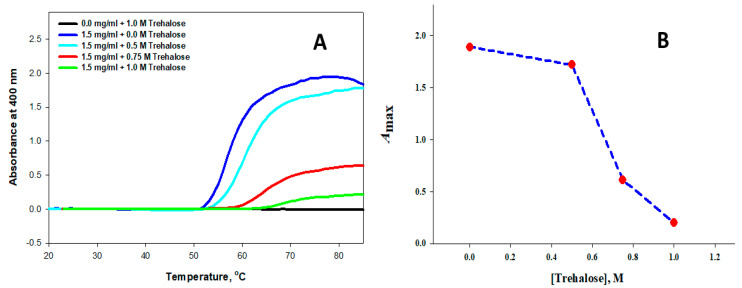
The thermal aggregation of α-LA in the presence of different concentrations of trehalose at pH 4.5. (**A**) Light Scheme 400. versus [Trehalose]. Each thermal aggregation profile was fitted according to Equation (1). (**B**) A plot of *A*_max_ (maximum absorbance) versus Trehalose (the molar concentration).

To evaluate the aggregation profile of the apo form of α-LA, thermal transition curves at a fixed concentration of the protein (1.5 mg mL^−1^) as used earlier [27] were measured in the presence of different molar concentrations of trehalose (0.5, 0.75 and 1.0 M) by following changes in absorbance at 400 nm (*A*_400_) in the temperature range 20–85 °C at pH 4.5. Figure 1A shows the results of these measurements. It should be noted that an increase in absorbance occurred due to an increase in the scattering of light by the presence of aggregates. It can be seen in Figure 1A that the *A*_440_ of the protein in the absence of trehalose increased at higher temperatures. This absorbance decreased as we increased the osmolyte concentration. At the highest osmolyte concentration (1 M) used in this study, the protein’s absorbance was reduced from 1.89 in the absence of osmolytes to 0.2 (see Figure 1A and Table 1). From the results shown in Figure 1A, it can be concluded that trehalose protected the protein from aggregation. Furthermore, protection from aggregation increased with an increase in the osmolyte concentration.

Equation (1) was used to analyze each curve shown in Figure 1A. For parameters *A*, *A*_max_, *T*_max_ and *b,* we utilized the dependence of *A*_400_ on the temperature to estimate the variable trehalose concentration. The results of this analysis of each thermal transition curve are given in Table 1. This Table also shows *T*i’s values, the point at which aggregation started, which was estimated using the relation (*T*_agg_ − 2*b*) [43]. It can be seen from Figure 1A and Table 1 that the initiation of the aggregation of the protein was delayed in the presence of trehalose, and this delay (*T*_i_) increased with an increase in the concentration of the osmolyte. For example, the aggregation delayed to 64 °C in the presence of 1 M trehalose instead of 53 °C in the absence of trehalose. Hence, it was visualized that 1 M trehalose showed the best inhibition of aggregation. How trehalose protected the protein from aggregation at higher temperatures could be understood by plotting *A*_max_, the maximum absorbance versus the concentration of trehalose, shown in Figure 1B. This Figure shows that aggregation was significantly reduced from 1.89 in the absence of trehalose to 0.2 in the presence of 1 M trehalose. Data are shown in Table 1 (also see Figure 1A,B) led us to conclude that a higher concentration of the osmolyte could protect proteins from aggregation at high temperatures. This observation supported earlier studies that indicated that trehalose stabilizes proteins by the mechanism of preferential hydration [29,44,45]. This osmolyte property would increase the lag period of the onset of aggregation with an increase in its concentration.

(1) $$A=Ao\;+\;\frac{Amax}{(1+e\left(-\left(T-Tagg\right)/b\right)\;}$$

where *A* is the absorbance at any temperature, *T*, *A_o_* is the absorbance of the initial baseline, *A_max_* is the absorbance of the final plateau line, *b* is the constant independent of *T* at a given wavelength, and osmolyte concentration and *A_agg_* is the temperature at 50% of the maximum absorbance occurrence.

### 2.2. Fluorescence Study

#### 2.2.1. Trehalose Effect on Thioflavin T Assay

**Figure 2:**
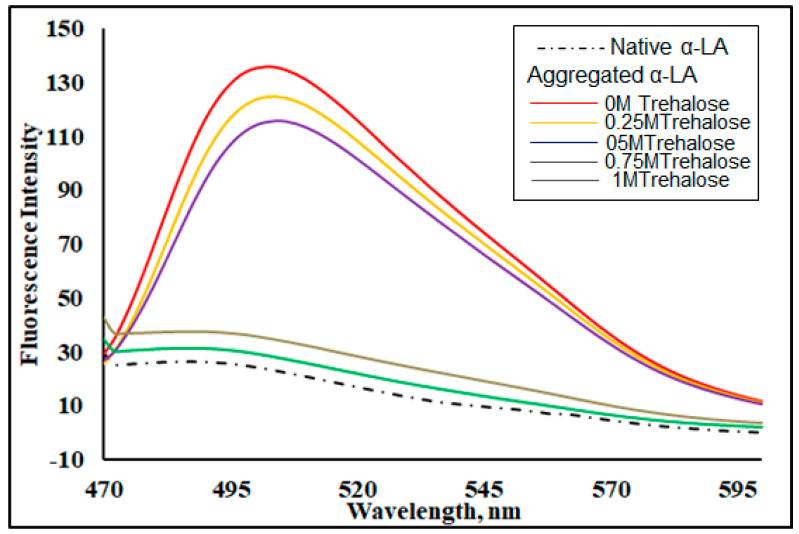
Thioflavin T (ThT) fluorescence intensity in the presence of the native apo-α-LA at 25 °C and thermally aggregated at 70 °C for 30 min in the presence of varying molar concentrations of trehalose (0, 0.25, 0.5, 0.75 and 1 M). All spectra were recorded at 25 °C.

Thioflavin T (ThT) is a characteristic dye that defines the composition of the cross-β-sheet responsible for the formation of amyloids or occasionally amorphous proteins in aggregates. As ThT interacts with amyloid fibrils, it exhibits a high fluorescence in the range of 480–500 nm after excitation at 450 nm [46,47]. The rise in the intensity of ThT fluorescence by several folds relative to that of the native protein suggests the existence of aggregates as ThT is known to bind to β-sheets extensively [47] Upon a thermally induced aggregation at 70 °C for 30 min, α-LA (1.5 mg mL^−1^) was observed to have a high ThT fluorescence relative to that of the native protein (Figure 2). Figure 2 shows the ThT fluorescence in the presence of varying concentrations of trehalose. It should be noted that the measurements of all spectra shown in this Figure were obtained at 25 °C. It should also be noted that the ThT fluorescence spectrum was subtracted from that of ThT in the presence of an aggregated protein with different concentrations of osmolytes. No blank was subtracted from the ThT spectrum in the presence of the native protein while the spectrum of ThT in the presence of the native protein was subtracted from each of the spectra of the aggregated α-LA in the presence of different concentrations of trehalose. Spectra of the respective trehalose + ThT were subtracted from the spectra of the aggregated protein in the presence of varying concentrations of trehalose. In fact, the fluorescence spectrum in the presence of trehalose were the differential spectra. It was evident that 1 M trehalose caused a maximum reduction in ThT fluorescence intensity. Interestingly, the spectrum in the presence of 1 M osmolyte was very close to that of the dye in the presence of the native protein. Thus, trehalose at 1 M concentration acted as the best inhibitor in preventing the aggregation of α-LA, whereas 0.25 M was the least effective.

#### 2.2.2. Intrinsic Fluorescence

**Figure 3:**
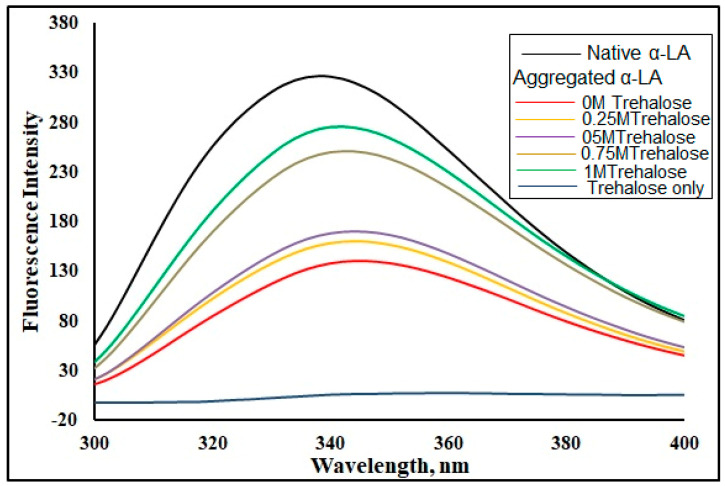
Intrinsic fluorescence spectra of the native apo-α-LA and aggregated α-LA in the absence and presence of different molar concentrations of trehalose at 25 °C.

A significant shift in the microenvironment surrounding the chromophore is indicative of the global transformation of the native protein to aggregate. The human α-LA contains three residues of tryptophan and four residues of tyrosine found in the protein at strategic positions [48] responsible for the cumulative intensity giving sharp absorption peaks at 280 nm. Intrinsic fluorescence emission spectra of the native α-LA and α-LA aggregated in the absence and presence of different molar concentrations of trehalose are shown in Figure 3. The native α-LA showed a maximum emission at around 337 nm when excited at 280 nm, a characteristic of the native α-LA. An aggregated α-LA solution showed a significant redshift and a visible decrease in Trp fluorescence than the native α-LA. This reduction in fluorescence, coupled with a significant redshift of 10 nm, indicated that the tryptophan residues’ burial changed to a more polar environment [49].

In the presence of trehalose, the aggregated α-LA solution displayed a decrease in intrinsic fluorescence in a concentration-dependent manner. In the presence of 1 M trehalose, the intrinsic fluorescence was nearly restored implying that 1 M trehalose acted as the best inhibitor in preventing the aggregation of α-LA. These observations were in accordance with the ThT fluorescence results and affirmed that 1 M trehalose acted as the best concentration for maximally inhibiting the formation of the α-LA aggregate.

#### 2.2.3. Rayleigh Scattering

**Figure 4:**
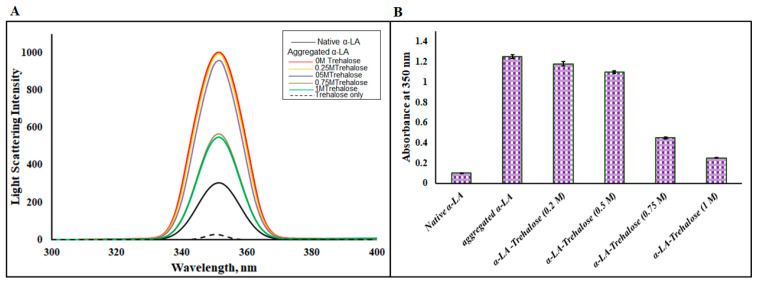
(**A**) Rayleigh scattering analysis of the native apo-α-LA and aggregated α-LA in the absence and presence of different molar concentrations of trehalose. (**B**) Turbidity measurements (absorbance at 350 nm) for the native α-LA and aggregated α-LA in the absence and presence of varying molar concentrations of trehalose. Error bars represent the standard errors of the mean estimated from at least three individual measurements.

Rayleigh–Tyndall scattering is a process that shows information in different directions about the dispersion induced by the samples. The radiation may arise from an individual molecule called Rayleigh scattering or colloidal suspension resulting from Tyndall scattering. Light scattering determination is a very sensitive technique for the identification of protein aggregation. Figure 4 depicts the degree of light scattering by native α-LA and aggregated α-LA in the absence and presence of a varying trehalose concentration (0.25, 0.5, 0.75 and 1 M). The native α-LA displayed the least light scattering, while the aggregated α-LA in the absence of trehalose displayed a massive increase in light scattering, which was indicative of aggregate formation [50]. In the presence of different molar concentrations of trehalose, α-LA displayed a substantial decrease in light dispersion; the spectrum in the presence of 1 M trehalose was very close to that of the native protein in the absence of osmolytes (Figure 4A). It is evident from Figure 4A that 0.25 M trehalose was the least effective in reducing light dispersion, whereas 1 M trehalose was found to be the most effective, implying that 1 M trehalose acted as the best concentration for maximally inhibiting the formation of α-LA aggregates. However, there was no significant light dispersion observed in the case of trehalose individually. These findings were consistent with previous studies confirming that 1 M trehalose serves as the most effective concentration in preventing α-LA aggregation.

### 2.3. Turbidity Assay

Turbidity refers to the haziness or cloudiness of a fluid caused by individual particles [49]. The aggregation propensity of α-LA was monitored spectrophotometrically at 350 nm in the absence and presence of trehalose’s varying molar concentration. The native protein showed a negligible or insignificant absorbance at 350 nm and, thus, a high absorbance at 350 nm was implicative of the formation of aggregates due to the scattering caused by larger aggregated particles [51]. Figure 4B shows that the thermally induced aggregation of α-LA (1.5 mg mL^−1^) in the absence of trehalose at 70 °C for 30 min showed a very high absorbance at 350 nm due to the existence of aggregates. There was an observed decrease in the absorbance at 350 nm with an increase in trehalose concentration; the maximum reduction was observed for 1 M trehalose. These observations were in line with earlier results that implied that the presence of 1 M trehalose prevented the aggregation of α-LA maximally; i.e., it acted as the best inhibitor in preventing the aggregation of α-LA.

### 2.4. TEM Analysis

**Figure 5:**
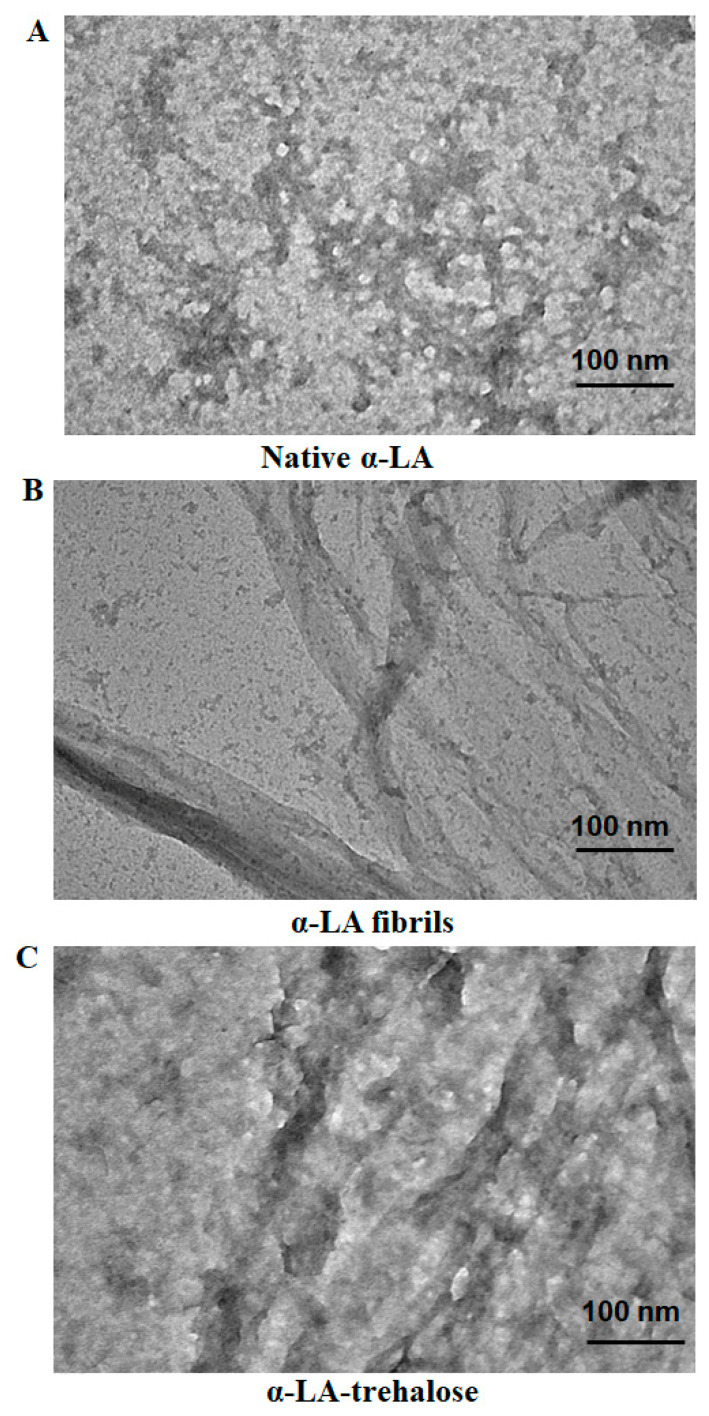
TEM images of (**A**) native α-LA, (**B**) α-LA fibrils and (**C**) α-LA-1M trehalose.

Microscopic studies further entrenched spectroscopic observations. Transmission electron microscopy was also deployed to check the effect of trehalose on the aggregation of apo-α-LA. Figure 5A,B show TEM images of the native α-LA and aggregated α-LA (1.5 mg mL^−1^) in the absence of trehalose (the formation of the aggregate occurred on heating the protein at 70 °C for 30 min), respectively. There existed abundant ribbon-like fibrils, reported earlier for aggregated α-LA [27]. Figure 5C shows TEM images of α-LA in the presence of 1 M trehalose. It can be seen in these Figures that 1 M trehalose inhibited α-LA aggregation. These microscopic observations were in line with earlier spectroscopic observations and affirmed that the presence of 1 M trehalose prevented the thermally induced aggregation of α-LA.

### 2.5. Molecular Docking Analysis

**Figure 6:**
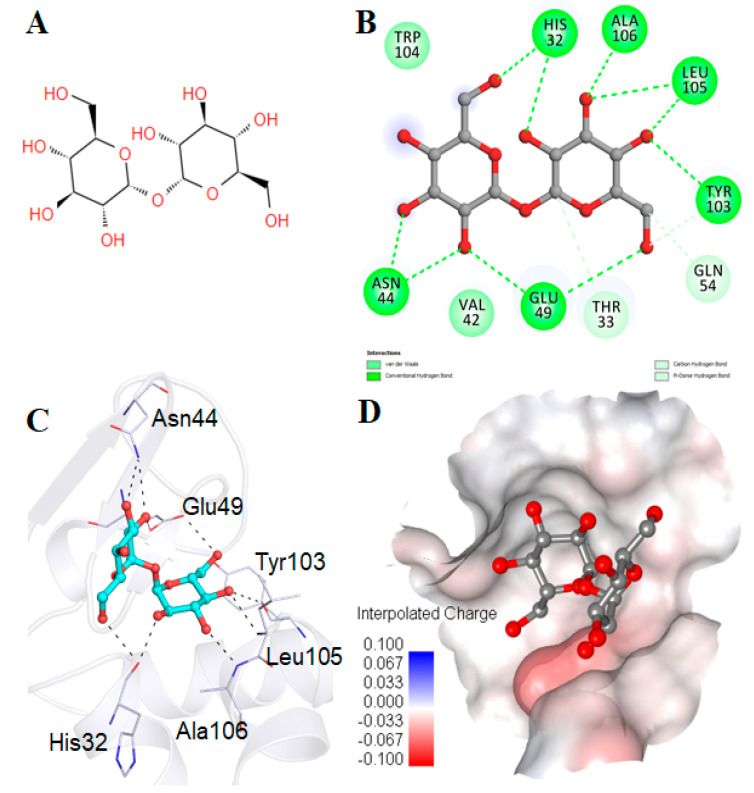
Presentation of the binding mode of trehalose to apo-α-LA. (**A**) 2D structural representation of trehalose. (**B**) 2D interaction of trehalose with the binding pocket residues of apo-α-LA. (**C**) α-LA complexed with trehalose showing hydrogen-bonded interactions with the residues of apo-α-LA binding pockets. (**D**) Charged surface view of α-LA complexed with trehalose.

After spectroscopic and microscopic observations, the next aim was to entrench our observations through silico analysis. Hence, molecular docking was performed to study the binding between α-LA and trehalose. Trehalose presented the docking score of −5.8 kcal mol^−1^ towards α-LA. It possessed the pKi (–log *K*i) and ligand efficiency values of 4.25 and 0.16 kcal mol^−1^ non-H atom^−1^, respectively. Interaction analysis of all possible docked conformers of trehalose was carried out to investigate their binding pattern and possible interactions towards the α-LA binding pocket. Trehalose preferentially occupied the binding site of α-LA with many interactions (Figure S2). Trehalose was present in the binding pocket cavity of α-LA and showed significant interactions with essential residues. Trehalose formed ten hydrogen bonds with six residues, His32, Asn44, Glu49, Tyr103, Leu105 and Ala106, along with a few van der Waals interactions (Figure 6B,C). An interpolated charged surface representation indicated that trehalose occupied the binding pocket cavity of α-LA with a virtuous complementarity fit (Figure 6D). The water replacement theory suggests that trehalose replaces hydrogen bonds between proteins and water and forms a hydrogen bond with the protein that balances out α-LA stability. Thus, these observations were in line with this theory, evident from the presence of ten hydrogen bonds. The study indicated that trehalose acted as a potent binding partner of α-LA and, hence, these in silico observations were as per in vitro assays affirming that trehalose acted as a potent inhibitor to prevent α-LA aggregation.

## 3. Materials and Methods

### 3.1. Materials

Lyophilized α-LA (holo) from bovines and trehalose were bought from Sigma–Aldrich Co. (St. Louis, MO, USA). Other chemicals were purchased from Merck, Germany. A millipore filter of pore size 0.22 µm was obtained from the Millipore Corporation Bangalore, India. A Whatman filter paper was bought from Whatman International Ltd. Twofold refined and de-ionized water from a Milli-Q^®^ UF-Plus filtration framework was utilized to prepare the buffer and other solutions. Figure S1 shows the structure of trehalose.

### 3.2. Protein Dialysis and Analytical Procedures

The required amount of holo-α-LA powder was dissolved in a 0.1 M KCl solvent solution. An apo form of α-LA was prepared by adding 5 mM (ethylene glycol-bis(β-aminoethyl ether)-N,N,N′,N′-tetraacetic acid) EGTA to a holo-α-LA (Ca^2+^ bound) solution. This solution of apo-α-LA was dialyzed against several changes of 0.1 M KCl solution at pH 7.0 and 4 °C. The dialysis tubing was prepared following the procedure of McPhie [52]. The dialyzed protein solution was filtered through millipore filters of pore size 0.22 µm.

### 3.3. Aliquots Preparation and Fibril Formation

The required amount of trehalose was dissolved in the 0.05 M sodium acetate buffer (pH 4.5), and this solution was filtered through a Whatman filter paper. The protein was incubated with different concentrations of trehalose before subjecting it to heat. A total of 30 min were given to provide equilibrium between the trehalose and the protein. The protein in the absence and presence of different trehalose concentrations was heated to 70 °C for 30 min to promote aggregation. The heated solution was cooled down to 25 °C and all spectroscopic measurements were done at 25 °C. All experiments were carried out in triplicate. A 1.55 M stock solution of trehalose, the most concentrated solution, was prepared in the buffer. The trehalose solution’s concentration was determined using the reported value of the refractive index [44,45,53]. The pre-incubated protein solution containing different trehalose amounts (0.25 M, 0.5 M, 0.75 M and 1.0 M) aggregated at 70 °C was used for further experiments. For comparison, apo-α-LA without trehalose was also incubated to be used as a control. Various biophysical techniques were used to characterize proteins under different solvent conditions.

### 3.4. UV-Vis Spectroscopy

Absorption measurements were carried out in a Jasco V-660 UV-Vis spectrophotometer equipped with a Peltier-type temperature controller. The absorption of protein solutions in the absence and presence of different trehalose concentrations (0.5, 0.75 and 1 M) was measured in the temperature range 20–85 °C. Equation (1) (shown in the Results and Discussion section) was used to analyze for aggregation parameters (*A*_0_, *A*_max_, *T*_agg_ and *b*) using a non-linear regression method.

### 3.5. Turbidity Assay

The apo-α-LA (1.5 mg mL^−1^) was incubated in different trehalose concentrations (0.25 M, 0.5 M, 0.75 M and 1.0 M) at 25 °C for 30 min to achieve an equilibrium. The aggregation was induced by heating the protein solution at 70 °C for 30 min. This heated sample was cooled down to 25 °C to measure turbidity at 350 nm by using a UV-Vis spectrophotometer (Jasco UV-660) as described earlier [27].The turbidity measurement was also made using a proper blank of a native apo-α-LA in a buffer (i.e., in the absence of trehalose).

### 3.6. Thioflavin T (ThT) Assay

The apo-α-LA solution containing a fixed concentration ratio of protein to ThT (1:10) was incubated with different trehalose concentrations at 25 °C for 30 min to achieve an equilibrium. The aggregation was induced in the solution by heating the protein samples in the presence of osmolytes at 70 °C for 30 min. The samples were cooled down to 25 °C, and fluorescence spectra measurements were taken in a Jasco FP-6200 spectrofluorometer using a 1 cm quartz cell. During these measurements, both the excitation and emission slit width were kept at 10 nm, and a 1 cm pathlength cell was used as described earlier [27]. All solutions contained 1.5 mg mL^−1^ protein and 15 mg mL^−1^ ThT [27]. To remove any insoluble particles, the solution was filtered with 0.22 μm before the measurements. ThT fluorescence contribution in the buffer was subtracted from each spectrum of the solution containing ThT, protein and trehalose.

### 3.7. Intrinsic Protein Fluorescence

The apo-α-LA (1.5 mg mL^−1^) was incubated with different trehalose concentrations (0.25 M, 0.5 M, 0.75 M and 1.0 M) at 25 °C for 30 min to achieve an equilibrium. The aggregation was induced in the solution by heating the protein samples in the presence of the osmolytes at 70 °C for 30 min. The samples were cooled down to 25 °C and excited at 280 nm with a 300–400 nm recording emission range. Spectra were measured in a Jasco FP-6200 spectrofluorometer (Tokyo, Japan) using a 1 cm pathlength quartz cell as described earlier [54].

### 3.8. Rayleigh Scattering

Rayleigh scattering measurements were performed in a Jasco FP-6200 spectrofluorometer (Tokyo, Japan) as reported earlier [47,55]. The apo-α-LA (1.5 mg mL^−1^) was incubated with different trehalose concentrations (0.25 M, 0.5 M, 0.75 M and 1.0 M) at 25 °C for 30 min to achieve an equilibrium. The aggregation was induced in the solution by heating the protein samples in the presence of trehalose at 70 °C for 30 min. The samples were cooled down to 25 °C, and the measurements were carried out.

### 3.9. Transmission Electron Microscopy

Transmission electron microscopy is a tool to provide an insight into the morphology of aggregates [56]. The apo-α-LA (1.5 mg mL^−1^) was subjected to thermal aggregation in the presence and absence of 1 M trehalose. The aggregates were placed on 400-mesh copper grids, covered with carbon stabilized formvar film and air-dried. The removal of excessive fluid was done after 2 min followed by the staining of the grid by using uranyl acetate. Samples were then air-dried and scanned in a TECNAI G2 20S-TWIN transmission electron microscope operating at an accelerating voltage of 80 kV.

### 3.10. Molecular Docking Analysis

The apo-α-LA structure was downloaded from the Protein Data Bank (PDB ID: 1F6S), and the trehalose structure was downloaded from the PubChem database (PubChem CID: 7427). The molecular docking of trehalose with α-LA was performed to predict their binding affinity and detailed interactions. The docking was performed using InstaDock, which uses QuickVina-W in docking calculations with a blind search space for the ligand [57,58]. PyMOL [59] and Discovery Studio Visualizer [60] were used for visualization and analysis.

We also calculated the inhibition constant [61], which is a negative decimal logarithm of the inhibition constant that comes from the ∆*G* parameter of the docking result. The p*Ki* value of both compounds was calculated while using the following formulae:

(2) $$\Delta G=RT\;\left(\mathrm{Ln}Ki_{\mathrm{pred}}\right)$$

(3) $$Ki_{\mathrm{pred}}=\;e^{\left(\Delta G/RT\right)}$$

(4) $$pK_{i}=\;-log(Ki_{\mathrm{pred}})$$

∆*G* was the binding affinity (kcal mol^−1^); *R* was the gas constant, 1.98 cal K^−1^ mol^−1^ [62]; *T* was the room temperature, 298.15 K; pred denotes predicted.

Ligand efficiency (LE) is one of the useful parameters used in lead selection by comparing average binding energy values per atom [61]. Here we calculated the LE of quercetin and naringenin while utilizing the following formula:

LE = −∆*G*/N (5)

LE was the ligand efficiency (kcal mol^−1^ non-H atom^−1^), ∆G was the binding affinity (kcal mol^−1^) and N was the number of non-hydrogen atoms in the ligand molecule.

## 4. Conclusions

UV-Vis spectroscopy, a ThT binding assay, intrinsic fluorescence, Rayleigh scattering and turbidity assay measurements showed that the presence of trehalose inhibited α-LA aggregation in a dosage-dependent manner with 1 M trehalose acting as the best concentration causing the maximal inhibition of protein aggregation. Microscopic techniques complemented these observations; TEM analysis suggested that the native α-LA was transformed into fibrils when subjected to thermal treatment and 1 M trehalose inhibited aggregation in αLA. Furthermore, in vitro and microscopic observations were supported by an in silico approach. Molecular docking studies suggested that trehalose as a potent binding partner of α-LA and hydrogen bonding were the key players in this interaction. Together with spectroscopic and microscopic observations, these observations affirmed that trehalose bonded with α-LA and the presence of 1 M trehalose prevented the aggregation of α-LA. This research gives evidence of the benefits of the naturally occurring sugars as inhibitors of amyloid fibril production and the possible use of naturally occurring sugar osmolytes for the therapeutic management of protein aggregation-related disorders.

## Figures and Tables

**Table 1:** Values of aggregation parameters obtained for temperature-dependent apo-α-LA aggregation at different concentrations of trehalose.

Concentration (mg mL^−1^)	*A*_o_	*A*_max_	*b*	*T*_agg_ (°C)	*T*_i_ (*T*_agg_ − 2*b*) (°C)
0.0	−0.010 ± 0.005	1.890 ± 0.009	2.610 ± 0.070	58.22 ± 0.08	53.02
0.5	−0.020 ± 0.006	1.727 ± 0.0105	2.788 ± 0.080	61.30 ± 0.08	55.72
0.75	−0.003 ± 0.002	0.617 ± 0.005	3.126 ± 0.100	65.87 ± 0.11	59.67
1.0	−0.002 ± 0.001	0.207 ± 0.002	2.899 ± 0.113	69.75 ± 0.13	64.17

## Data Availability

Not applicable.

## Supplementary Materials

The following are available online at https://www.mdpi.com/2218-273X/11/3/414/s1, Figure S1: Structure of trehalose, Figure S2: Diagrammatic representation of trehalose binding with alpha-lactalbumin with significant interactions offered Table S1: Different Physical and Chemical Properties of Trehalose.
