# Supplementary material for: Trehalose Restrains the Fibril Load towards α-Lactalbumin Aggregation and Halts Fibrillation in a Concentration-Dependent Manner
Source: Biomolecules. 2021 Mar 11;11(3):414. doi: 10.3390/biom11030414 (PMC8001226; doi:10.3390/biom11030414)
Supplement: Supplementary file 1 [file biomolecules-11-00414-s001.pdf]

Supplementary Materials

Trehalose restrains fibril load towards α-lactalbumin aggregation and halts fibrillation in a concentration-dependent manner

Sania Bashir [1], Ishfaq Ahmad Ahanger[1,2], Anas Shamsi [1,3], Mohamed F. Alajmi[4], Afzal Hussain[4], Hani Chaudhary[5], Faizan Ahmad [1], Md. Imtaiyaz Hassan [1] and Asimul Islam [1*]

[1]Centre for Interdisciplinary Research in Basic Sciences, Jamia Millia Islamia, Jamia Nagar, New Delhi 110025, India.
[2]Department of Chemistry, Biochemistry and Forensic Science, Amity School of Applied Sciences, Amity University Haryana-122 413
[3] Centre of Medical and Bio-Allied Health Sciences Research, Ajman University, UAE.
[4]Department of Pharmacognosy College of Pharmacy, King Saud University, Riyadh, Saudi Arabia.
[5] Department of Biochemistry, Faculty of Sciences, Cancer and Mutagenesis Unit, King Fahd Medical Research Center, King Abdulaziz University, Jeddah 21589, Saudi Arabia.
*Correspondence: *aislam@jmi.ac.in* (A.I.)

***To whom all correspondence should be addressed,**

Asimul Islam, Ph.D.
Centre for Interdisciplinary Research in Basic Sciences
Jamia Millia Islamia, Jamia Nagar
New Delhi 110025, INDIA
Cell: +91-9312812007
E-mail: aislam@jmi.ac.in

Table S1. Different Physical and Chemical Properties of Trehalose

Parameter	Value
Chemical formula	$C_{12}H_{22}O_{11}$ (anhydrous)<br>$C_{12}H_{22}O_{11}\cdot 2H_2O$ (dihydrate)
Molecular weight	342.3 g mol^{-1} (anhydrous)<br>378.3 g mol^{-1} (dihydrate)
Appearance	White, odourless powder
Sweetness (relative)	45% that of sucrose
Polymorphs	Two crystalline forms: anhydrous (T_α, T_β) and dihydrate (T_h), one well-defined amorphous form
Chemical nature	Nonreducing disaccharide (α-D-glucopyranosyl-α-D-glucopyranoside)
Relative density	1.22 g cm^{-3} (at 25°C and weight fraction = 0.5)
Glass transition temperature	117°C
Thermostability	>99% (120°C/90 min)
Melting point	203°C (anhydrous)<br>97°C (dihydrate)
Heat of fusion	53.4 kJ mol^{-1} (anhydrous)<br>57.8 kJ mol^{-1} (dihydrate)
Hygroscopicity	None at <90% RH
Solubility in water	68.9 g/100 g (at 20°C)
Freezing temperature (100 mg mL^{-1} water)	−197°C
Optical rotation	$[\alpha]_D = +178$°C
Viscosity	8.2 cp (at 40°C and weight fraction = 0.5)
Chemical reactivity	Does not usually react with proteins
Regulatory status	GRAS (generally regarded as safe) by US FDA

RH, relative humidity.

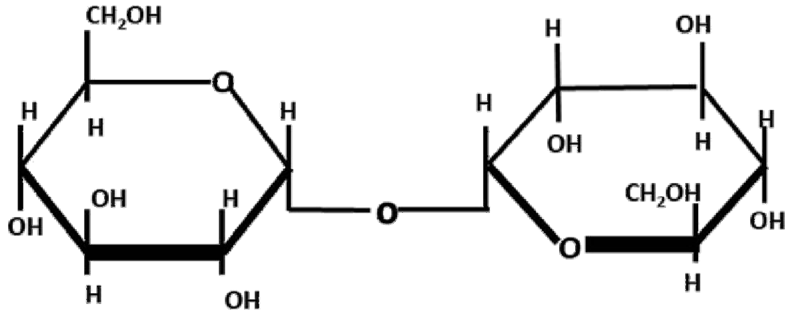

Figure S1. Structure of trehalose.

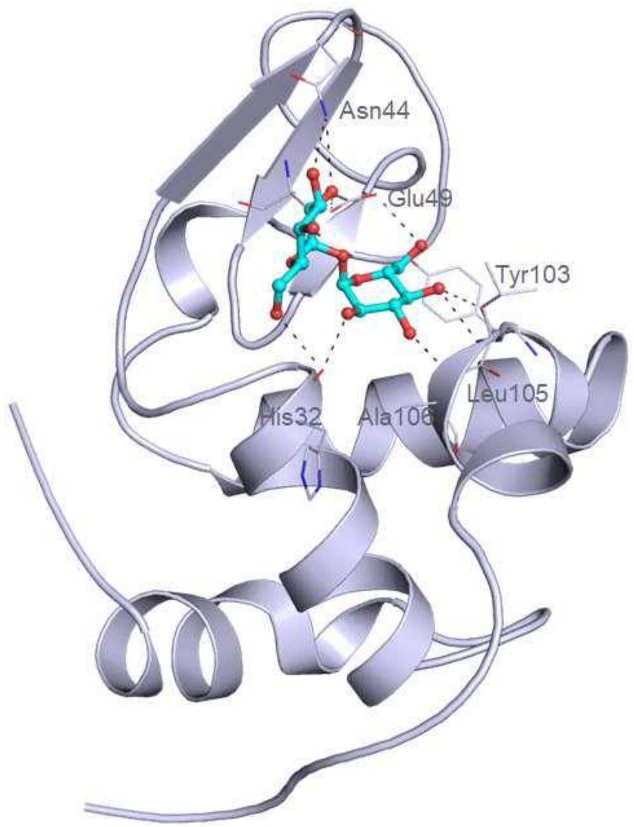

Figure S2. Diagrammatic representation of trehalose binding with alpha-lactalbumin with significant interactions offered.
